# Weighted multimodal family of distributions with sine and cosine weight functions

**DOI:** 10.1016/j.heliyon.2020.e04757

**Published:** 2020-08-28

**Authors:** Ayman Alzaatreh, Jaber Kazempoor, Adel Ahmadi Nadi

**Affiliations:** a Department of Mathematics and Statistics, American University of Sharjah, Sharjah, United Arab Emirates; b Department of Statistics, Faculty of Mathematical Sciences, Ferdowsi University of Mashhad, Islamic Republic of Iran

**Keywords:** Mathematics, Moments, Characteristic function, Even probability density function, Parseval's identity

## Abstract

In this paper, the moment of various types of sine and cosine functions are derived for any random variable. For an arbitrary even probability density function, the sine and cosine moments are used to define new families of univariate multimodal probability density and their corresponding characteristic functions. For illustration, two weighted multimodal generalizations of the *t* distribution are investigated. Furthermore, a method of calculating some interesting improper integrals is also presented. Finally, an explicit expression of the probability density function of the sum of independent *t*-distributed random variables with odd degrees of freedom is derived.

## 1 Introduction

A multimodal distribution is a probability distribution with at least two modes (local maxima). Multimodal distributions are popular in modelling various types of data. There exist many empirical distributions in practice with multimodal form. For this reason, researchers proposed several methods for constructing multimodal family of distributions. A common way of constructing a proper multimodal distribution is by utilizing mixtures of some known distributions. In this case, the new mixture probability density function (pdf) is a weighted sum of known pdfs as follows; $g(t)=\sum_{i=1}^{n}\alpha_{i}g_{i}(t)$, $0\leq \alpha_{i}\leq 1$, i=1,2,…,n, with $\sum_{i=1}^{n}\alpha_{i}=1$. The choice of the weights, $\alpha_{i}$, plays a main role in modelling data using the mixture pdf g(t). For more information, the reader is referred to [1], [2] and the references cited therein.

Another way of modelling univariate multimodal data is by using weighted distributions. Fisher [3], brought the notion of weighted distributions in order to model ascertainment biases. Later, Rao [4], used weighted distributions in an exceedingly unifying theory for issues related when observations fall into non-experimental, non-replicated and non-random way. The weighted distribution, g(x), is defined as $g(x)=\frac{w(x)f(x)}{E[w(X)]}$, where f(.) is a pdf of the random variable *X* and w(.) is a positive function. Here, *E* stands for the mathematical expectation where $E(w(X))=\int_{R}w(x)f(x)dx$. Some common weighted functions, for example, are $w(x)=F^{i-1}(x){\overset{¯}{F}}^{n-i}(x)$ and w(x)=x which respectively correspond to the *i*-th order statistics arising from *n* independent and identically random variables with absolutely continuous cumulative density function (cdf) F(.) ([5, Page 10]), and length biased pdfs [6]. For comprehensive discussions about weighted distributions, readers are referred to [7] and [8].

In this paper, some new family of multimodal distributions are proposed and discussed. These families are constructed in two ways. The first method is by applying weighted distribution technique with weight functions involving cosine and sine. This technique requires calculating the moments of sine and cosine functions which is also discussed in this paper. The second method is defining new family of distributions based on sine and cosine functions. In this method, the constructed family of distributions is multimodal because of the fact that sine and cosine functions have the multimodality property.

The rest of the paper is organized as follows. In section 2, moment of sine and cosine functions of any order is derived. Also new family of weighted multimodal distributions are proposed. Some applications of the main results are discussed in Section 3. In particular, we present some examples of new weighted distributions. Also, we derive explicit forms of some complex improper integrals. Furthermore, we derive an explicit expression of the probability density function of sum of independent *t*-distributed random variables with odd degrees of freedom in Section 3.

## 2 Main results

In this section, the expectation of $e^{-iuX}cos^{n}(rX),u\in R$ is discussed for any arbitrary continuous random variable *X*. Note that

$$\left|\;\int_{-\infty }^{\infty }e^{-iux}\cos^{n}(rx)f_{X}(x)dx\right|\leq \int_{-\infty }^{\infty }\left|e^{-iux}\cos^{n}(rx)\right|f_{X}(x)dx\leq \int_{-\infty }^{\infty }f_{X}(x)dx=1,\;i^{2}=-1,$$

is valid for any continuous random variable *X* with pdf $f_{X}(.)$. Therefore, if u=0, the expectation of $\cos^{n}(rX)$ exists for any values of *n* and *r*. The same result can be obtained for the moment of sine functions.

Let $\psi_{X}:R⟶C$ be the characteristic function of *X* defined by $\psi_{X}(t)=E[e^{itX}]=\int_{-\infty }^{\infty }e^{itx}dF_{X}(x)$, where $F_{X}(.)$ is the cumulative distribution function of *X*. $\psi_{X}$ enjoys several important properties such as its existence. For other properties, the reader is referred to [9] and [7]. Now, for a random variable *X* whose pdf is symmetric about 0, it is easy to see that $\psi_{X}(t)=E(\cos(tX))$. Consequently, $E{\left(\cos(mX)\right)}^{n}$ and $E{\left(\sin(mX)\right)}^{n}$ can be derived using some trigonometric identities ([7]). However, it is clear that the indirect computations of high order moments of sine and cosine functions using trigonometric identities are time-consuming and not attractive. Also, it requires the symmetry condition. For these reasons, the following result presents direct formulas for computing the moment of sine and cosine functions for any continuous random variable.

Lemma 1 *Let X be a continuous random variable with cumulative density and characteristic functions* $F_{X}(.)$ *and* $\psi_{X}(.)$ *respectively, then for* r,u∈R,n∈N *we have*

$$\int_{-\infty }^{\infty }e^{-iut}{\left[\cos(tr)\right]}^{2n}dF_{X}(t)=\frac{\sum_{w=0}^{2n}\left(\begin{array}{c} 2n\\ w \end{array}\right)\psi (2r(w-n)-u)}{4^{n}},$$

$$\int_{-\infty }^{\infty }e^{-iut}{\left[\cos(tr)\right]}^{2n-1}dF_{X}(t)=\frac{2\sum_{w=0}^{2n-1}\left(\begin{array}{c} 2n-1\\ w \end{array}\right)\psi (2r(w-n)+r-u)}{4^{n}}$$

*and*

$$\int_{-\infty }^{\infty }e^{-iut}{\left[\sin(tr)\right]}^{2n}dF_{X}(t)=\psi (-u)+\sum_{k=1}^{n}\left(\begin{array}{c} n\\ k \end{array}\right){\left(-\frac{1}{4}\right)}^{k}\sum_{w=0}^{2k}\left(\begin{array}{c} 2k\\ w \end{array}\right)\psi (2r(w-k)-u),$$

Proof Let $Y_{1},Y_{2},\ldots ,Y_{m}$ be independent and identically distributed random variables such that

$$P(Y_{j}=r)=P(Y_{j}=-r)=\frac{1}{2},r\neq 0.$$

By using the Parseval's identity ([9, Page 172]) we get

$$\int_{-\infty }^{\infty }e^{-iut}\psi_{\sum_{j=1}^{m}Y_{j}}(t)dF_{X}(t)=\int_{-\infty }^{\infty }\psi_{X}(t-u)dF_{\sum_{j=1}^{m}Y_{j}}(t).$$

It is easy to see that

$$\psi_{\sum_{j=1}^{m}Y_{j}}(t)={\left[\cos(tr)\right]}^{m},$$

and using the fact that $\frac{\sum_{j=1}^{m}Y_{j}+mr}{2r}∼Binomial(m,1/2)$, we have

$$F_{\sum_{j=1}^{m}Y_{j}}(t)=2^{-m}\sum_{j=-mr,-mr+2r,-mr+4r,\ldots }^{mr}\left(\begin{array}{c} m\\ \frac{1}{2}(m+\frac{j}{r}) \end{array}\right)I(j\leq t),$$

or

$$F_{\sum_{j=1}^{m}Y_{j}}(t)=2^{-m}\sum_{j=0}^{m}\left(\begin{array}{c} m\\ j \end{array}\right)I(j\leq \frac{t+mr}{2r}).$$

Finally, one can get the results by using the fact that

$${\left[\sin(tr)\right]}^{2n}={\left[1-{\left(\cos(tr)\right)}^{2}\right]}^{n}=1+\sum_{k=1}^{n}\left(\begin{array}{c} n\\ k \end{array}\right){\left(-1\right)}^{k}{\left(\cos(tr)\right)}^{2k}.$$

 □

Note that a similar formula for odd power of sin⁡x can also be derived in Theorem 1 by using ${\left[\sin(tr)\right]}^{2n-1}=1+\sum_{k=1}^{\infty }\left(\begin{array}{c} n-1/k\\ k \end{array}\right){\left(-1\right)}^{k}{\left(\cos^{2}(tr)\right)}^{k}$. However, the formula is not useful in this paper. Next corollary defines interesting families of multimodal distributions with closed form characteristic functions.

Corollary 1 *Let X be an absolutely continuous random variable with cumulative density and characteristic functions* $F_{X}(.)$ *and* $\psi_{X}(.)$ *respectively, then*

(1) $$g(x)=\frac{4^{n}{\left[\cos(xr)\right]}^{2n}f_{X}(x)}{\sum_{w=0}^{2n}\left(\begin{array}{c} 2n\\ w \end{array}\right)\psi_{X}(2r(w-n))},$$

*is a pdf with characteristic function is given by*

(2) $$\psi (t)=\frac{\sum_{w=0}^{2n}\left(\begin{array}{c} 2n\\ w \end{array}\right)\psi_{X}(2r(w-n)+t)}{\sum_{w=0}^{2n}\left(\begin{array}{c} 2n\\ w \end{array}\right)\psi_{X}(2r(w-n))}.$$

*Moreover*

(3) $$g(x)=\frac{{\left[\sin(xr)\right]}^{2n}f_{X}(x)}{1+\sum_{k=1}^{n}\left(\begin{array}{c} n\\ k \end{array}\right){\left(-\frac{1}{4}\right)}^{k}\sum_{w=0}^{2k}\left(\begin{array}{c} 2k\\ w \end{array}\right)\psi_{X}(2r(w-k))},$$

*is a pdf with characteristic function is given by*

(4) $$\psi (t)=\frac{\psi_{X}(t)+\sum_{k=1}^{n}\left(\begin{array}{c} n\\ k \end{array}\right){\left(-\frac{1}{4}\right)}^{k}\sum_{w=0}^{2k}\left(\begin{array}{c} 2k\\ w \end{array}\right)\psi_{X}(2r(w-k)+t)}{1+\sum_{k=1}^{n}\left(\begin{array}{c} n\\ k \end{array}\right){\left(-\frac{1}{4}\right)}^{k}\sum_{w=0}^{2k}\left(\begin{array}{c} 2k\\ w \end{array}\right)\psi_{X}(2r(w-k))}.$$

Remarks

I. If n=0, then the pdfs in (1) and (3) reduce to the baseline distribution $f_{X}(x)$. Therefore, (1) and (3) can be considered as multimodal generalizations of $f_{X}(x)$.
II. The families of distributions defined in equations (1) and (3) can be viewed as weighted family of distributions of the form $g(x)=\frac{w(x)f_{X}(x)}{E(w(X))}$ with weight functions, respectively, are ${\left[\cos(rx)\right]}^{2n}$ and ${\left[\sin(rx)\right]}^{2n}$.
III. If *X* has a symmetric pdf, then the characteristic function is real and even function [9, Page 165]. Moreover, the even power moments of sine and cosine functions are given by
  $$\int_{-\infty }^{\infty }{\left[\cos(tr)\right]}^{2n}dF_{X}(t)=\frac{\left(\begin{array}{c} 2n\\ n \end{array}\right)+2\sum_{w=1}^{n}\left(\begin{array}{c} 2n\\ n-w \end{array}\right)\psi (2rw)}{4^{n}},$$
  and
  $$\int_{-\infty }^{\infty }{\left[\sin(tr)\right]}^{2n}dF_{X}(t)=1+\sum_{k=1}^{n}\left(\begin{array}{c} n\\ k \end{array}\right)\left(\begin{array}{c} 2k\\ k \end{array}\right){\left(-\frac{1}{4}\right)}^{k}+2\sum_{k=1}^{n}\left(\begin{array}{c} n\\ k \end{array}\right){\left(-\frac{1}{4}\right)}^{k}\sum_{w=1}^{k}\left(\begin{array}{c} 2k\\ k-w \end{array}\right)\psi (2rw).$$

## 3 Applications

In this section, we present some applications of the results in Section 2. In particular, Example 1 shows how some complex improper integrals can be calculated easily. Example 2 presents an example of a weighted multimodal distribution generated using the *t* distribution as a baseline.

It is interesting to note that the characteristic function of some random variables has been used to solve some difficult integrals. For example, Gut [9] utilized the characteristic function to solve $\int_{0}^{\infty }\frac{\sin\;x}{x}dx$ and $\int_{0}^{\infty }\frac{\sin^{2}x}{x^{2}}dx$. In Example 1, we show how the characteristic function can be used easily to solve some other integrals. Moreover an interesting explicit expression for solving the improper integral $\int_{0}^{\infty }\frac{\sin^{m}(x)}{x^{m}}dx$ is also derived for any integer *m*. First we state the following theorem from [10] with some minor changes referred as the theory of positive definite densities.

Theorem 1 *Let* $\psi_{X}(t)$ *be a real characteristic function such that* $\int_{-\infty }^{\infty }|\psi_{X}(t)|dt<\infty$*, then X has an absolutely continuous distribution with a bounded, symmetric pdf* $f_{X}=F_{X}^{\prime }$*, given by*

$$f_{X}(x)=\frac{1}{2\pi }\int_{-\infty }^{\infty }e^{itx}\psi_{X}(t)dt.$$

*Moreover* $g_{X}(x)=\frac{\psi_{X}(x)}{2\pi f_{X}(0)}$ *is a symmetric pdf with symmetric characteristic function* $\psi^{⁎}$*, given by*

$$\psi_{X}^{⁎}(t)=\frac{f_{X}(t)}{f_{X}(0)}.$$

Example 1 **Explicit formulas of some improper integrals**

In this example, we show how Lemma 1 and Theorem 1 can be used in order to derive closed form solutions for some interesting integrals such as $\int_{0}^{\infty }\frac{\sin^{m}(x)}{x^{m}}dx,m\in N$.

Let the random variable *X* follow the Triangular distribution with pdf given by

$$f_{X}(x)=\frac{c-|x|}{c^{2}}I_{\left[-c,c\right]}(x).$$

It is easy to see that

$$\psi_{X}(t)=2\frac{1-\cos(ct)}{c^{2}t^{2}}I_{\left[-\infty ,\infty \right]}(t).$$

By using Corollary 1 and Lemma 1, we get the following two pdfs

(5) $$\frac{4^{n}{\left[\cos(rx)\right]}^{2n}\frac{c-|x|}{c}}{\left(\begin{array}{c} 2n\\ n \end{array}\right)+2\sum_{w=1}^{n}\left(\begin{array}{c} 2n\\ w \end{array}\right)\psi (2rw)}$$

and

(6) $$\frac{4^{n}{\left[\cos(rx)\right]}^{2n-1}\frac{c-|x|}{c}}{2\sum_{w=0}^{2n-1}\left(\begin{array}{c} 2n-1\\ w \end{array}\right)\psi (2r(w-n)+r)}.$$

The corresponding characteristic functions, respectively, given by

(7) $$\frac{\sum_{w=0}^{2n}\left(\begin{array}{c} 2n\\ w \end{array}\right)\psi (2r(w-n)+t)}{\left(\begin{array}{c} 2n\\ n \end{array}\right)+2\sum_{w=1}^{n}\left(\begin{array}{c} 2n\\ w \end{array}\right)\psi (2rw)}$$

and

(8) $$\frac{\sum_{w=0}^{2n-1}\left(\begin{array}{c} 2n-1\\ w \end{array}\right)\psi (2r(w-n)+r+t)}{\sum_{w=0}^{2n-1}\left(\begin{array}{c} 2n-1\\ w \end{array}\right)\psi (2r(w-n)+r)}.$$

Corollary 2 *The following explicit formulas can be obtained.*

(i) *From equations* (5) *and* (7) *and* Theorem 1*, we get the following result*
  (9) $$c(c-|x|)\pi 4^{n}{\left[\cos(rx)\right]}^{2n}=\sum_{w=0}^{2n}\left(\begin{array}{c} 2n\\ w \end{array}\right)\int_{-\infty }^{\infty }e^{itx}\frac{1-\cos(c(2r(w-n)+t))}{{\left(2r(w-n)+t\right)}^{2}}dt.$$
(ii) *From equations* (6) *and* (8) *and* Theorem 1*, we get the following result*
  (10) $$c(c-|x|)\pi 4^{n}{\left[\cos(rx)\right]}^{2n-1}\;\;=\sum_{w=0}^{2n-1}\left(\begin{array}{c} 2n-1\\ w \end{array}\right)\int_{-\infty }^{\infty }e^{itx}\frac{1-\cos(c(2r(w-n)+r+t))}{{\left(2r(w-n)+r+t\right)}^{2}}dt.$$

On setting n=x=0 and c=1 in equation (9), we get

$$\pi =\int_{-\infty }^{\infty }\frac{1-\cos t}{t^{2}}dt$$

and hence

$$\frac{\pi }{2}=\int_{0}^{\infty }\frac{\sin^{2}(t)}{t^{2}}dt.$$

Now, for the general case, $\int_{0}^{\infty }\frac{\sin^{m}(x)}{x^{m}}dx,m\in N$, consider $X_{1},X_{2},\ldots ,X_{m}$ to be independent and uniformly distributed random variables on (−1,1). Then the random variable $Y=\sum_{j=1}^{m}X_{j}$ has the characteristic function $\psi_{\sum_{j=1}^{m}X_{j}}(t)=\frac{\sin^{m}(t)}{t^{m}}$ and equidistributed with 2Z−m, where *Z* follows the Irwin–Hall distribution [11]. Therefore,

(11) $$f_{\sum_{j=1}^{m}X_{j}}(y)=\frac{\sum_{k=0}^{m}{\left(-1\right)}^{k}\left(\begin{array}{c} m\\ k \end{array}\right){\left(\frac{y+m}{2}-k\right)}^{m-1}\text{sgn}\left(\frac{y+m}{2}-k\right)}{4(m-1)!},-m\leq y\leq m,$$

where sgn(.) is the sign function defined as

$$\text{sgn}(x)=\left\{ \begin{array}{cc} -1, & \;x<0\\ 0, & \;x=0\\ +1, & \;x>0. \end{array}\right.$$

Now, from equation (11), Corollary 1 and Lemma 1, it is straightforward to see that

$$g_{Y}(y)=\frac{4^{n-1}{\left[\cos(ry)\right]}^{2n}\sum_{k=0}^{m}{\left(-1\right)}^{k}\left(\begin{array}{c} m\\ k \end{array}\right){\left(\frac{y+m}{2}-k\right)}^{m-1}\text{sgn}\left(\frac{y+m}{2}-k\right)}{(m-1)!\left[\left(\begin{array}{c} 2n\\ n \end{array}\right)+2\sum_{w=1}^{n}\left(\begin{array}{c} 2n\\ n-w \end{array}\right){\left(\frac{\sin(2rw)}{2rw}\right)}^{m}\right]},$$

is a probability density function with the following characteristic function

$$\phi_{Y}(t)=\frac{\left(\begin{array}{c} 2n\\ n \end{array}\right)+\sum_{w=1}^{n}2\left(\begin{array}{c} 2n\\ w-n \end{array}\right){\left(\frac{\sin(2rw+t)}{2rw+t}\right)}^{m}}{\left(\begin{array}{c} 2n\\ n \end{array}\right)+\sum_{w=1}^{n}2\left(\begin{array}{c} 2n\\ w-n \end{array}\right){\left(\frac{\sin(2rw)}{2rw}\right)}^{m}}.$$

Or by symmetry,

$$\phi_{Y}(t)=\frac{\sum_{w=0}^{2n}\left(\begin{array}{c} 2n\\ w \end{array}\right){\left(\frac{\sin(2r(w-n)+t)}{2r(w-n)+t}\right)}^{m}}{\sum_{w=0}^{2n}\left(\begin{array}{c} 2n\\ w \end{array}\right){\left(\frac{\sin(2r(w-n))}{2r(w-n)}\right)}^{m}}.$$

Now by using Theorem 1, we get the following result

(12) $$\sum_{w=0}^{2n}\left(\begin{array}{c} 2n\\ w \end{array}\right)\int_{-\infty }^{\infty }e^{ity}{\left(\frac{\sin(2r(w-n)+t)}{2r(w-n)+t}\right)}^{m}dt=\frac{\pi 2^{2n-1}{\left[\cos(ry)\right]}^{2n}\sum_{k=0}^{m}{\left(-1\right)}^{k}\left(\begin{array}{c} m\\ k \end{array}\right){\left(\frac{y+m}{2}-k\right)}^{m-1}\text{sgn}\left(\frac{y+m}{2}-k\right)}{(m-1)!}.$$

Corollary 3 *Setting* n=0 *and* y=0 *in equation* (12)*, we get*

$$\int_{0}^{\infty }\frac{\sin^{m}(x)}{x^{m}}dx=\frac{\pi }{4}\frac{\sum_{k=0}^{m}{\left(-1\right)}^{k}\left(\begin{array}{c} m\\ k \end{array}\right){\left(\frac{m}{2}-k\right)}^{m-1}\text{sgn}\left(\frac{m}{2}-k\right)}{(m-1)!}.$$

In Table 1, we used the explicit formula in Corollary 3 to compute the exact value of $\int_{0}^{\infty }\frac{\sin^{m}(x)}{x^{m}}dx$ for m=1,2,…,9.

**Table 1:** Exact value of $\int_{0}^{\infty }\frac{\sin^{m}(x)}{x^{m}},\;\;m=1,2,\ldots ,9$.

m	1	2	3	4	5	6	7	8	9
Value	$\frac{1}{2}\pi$	$\frac{1}{2}\pi$	$\frac{3}{8}\pi$	$\frac{1}{3}\pi$	$\frac{115}{384}\pi$	$\frac{11}{40}\pi$	$\frac{5887}{23040}\pi$	$\frac{151}{630}\pi$	$\frac{259723}{1146880}\pi$

Example 2 **Sine and cosine multimodal generalizations of t-distribution**

In this example, we use Corollary 1 in order to generate weighted multimodal extensions of a baseline distribution $f_{X}(x)$. In particular, we use the standard *t* distribution as a baseline for illustration.

The student *t* distribution with *s* degree of freedom has the following symmetric pdf

(13) $$f_{X}(x,s)=\frac{\Gamma \left(\frac{s+1}{2}\right)}{\sqrt{s\pi }\Gamma \left(\frac{s}{2}\right)}{\left(1+\frac{x^{2}}{s}\right)}^{-\frac{s+1}{2}}.$$

The corresponding integral form of the characteristic function [12] is given by

(14) $$\psi_{X}(t,s)=\frac{{\left(2\sqrt{s}\right)}^{s}}{\Gamma (s)}\int_{R}e^{-\sqrt{s}(2x+|t|)}{\left(x(x+|t|)\right)}^{\frac{s-1}{2}}dx.$$

On using Corollary 1 and equation (14), we get the following two weighted probability distributions with pdfs given by

(15) $$g_{1}(x,s)=\frac{4^{n}{\left[\cos(rx)\right]}^{2n}f_{X}(x,s)}{\sum_{w=0}^{2n}\left(\begin{array}{c} 2n\\ w \end{array}\right)\psi_{X,s}(2r(w-n))},$$

and

(16) $$g_{2}(x,s)=\frac{{\left[\sin(rx)\right]}^{2n}f_{X}(x,s)}{1+\sum_{k=1}^{n}\left(\begin{array}{c} n\\ k \end{array}\right){\left(-\frac{1}{4}\right)}^{k}\sum_{w=0}^{2k}\left(\begin{array}{c} 2k\\ w \end{array}\right)\psi_{X}(2r(w-k),s)}.$$

**Figure 1:**
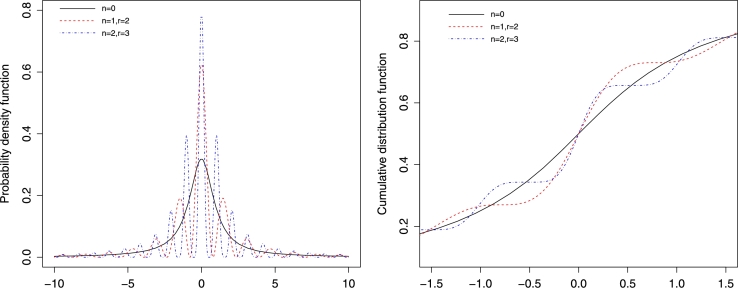
PDF plots of the multimodal cosine form of the t-distribution with degree of freedom *s* = 1.

**Figure 2:**
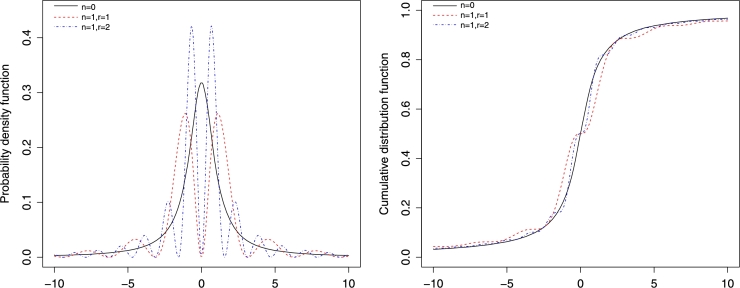
PDF plots of multimodal sine form of t-distribution with degree of freedom *s* = 1.

Plots of the pdf and the cdf for cosine *t* distribution [equation (15)] and sine *t* distribution [equation (16)] are, respectively, depicted for various parameter values in Figs. 1 and 2.

Example 3 **Further discussions on *t* distribution with odd degree of freedom**

The pdf of *t* distribution with even degree of freedom can be approximated by using the nearest odd degree of freedom. The characteristic function of *t* distribution has been investigated by many authors. An integral form of the characteristic function of *t* distribution was derived by Dreier [12] and recently Guant [13] presented a simple derivation of this function. More details about the characteristic function of the *t* distribution can be found in [12] and [13].

In this example, we present a derivation of the characteristic function of the *t* distribution with odd degree of freedom. Also, we derive an explicit form of the pdf of sum of independent *t* distributed random variables with odd degree of freedom.

A recurrence formula for the characteristic function of *t* distribution with odd degree of freedom was derived by Mitra [14] as follows

(17) $$\psi (t,2m-1)=e^{-|t\sqrt{2m-1}|}\sum_{j=0}^{m-1}c_{j,m-1}|t\sqrt{2m-1}{|}^{j},$$

where $c_{0,m}=c_{1,m}=1$, $c_{m-1,m}=\frac{1}{1\times 3\ldots (2m-5)\times (2m-3)}$, and $c_{j,m}=\frac{c_{j-1,m-1}+(2m-3-j)c_{j,m-1}}{2m-3},1\leq j\leq m-1$. For more details, see also [15, Chapter 28] and [16, Chapter 3]. Next, we derive an explicit form for the characteristic function of *t* distribution with odd degree of freedom.

Theorem 2 *The characteristic function of the t distribution in* (13) *with* 2m−1,m∈N *degree of freedom is given by*

(18) $$\psi (t,2m-1)=e^{-|\sqrt{2m-1}t|}\sum_{j=0}^{m-1}\frac{\left(\begin{array}{c} 2m-j-2\\ m-1 \end{array}\right)}{\left(\begin{array}{c} 2m-2\\ m-1 \end{array}\right)}\frac{{\left(2\sqrt{2m-1}t\right)}^{j}}{j!}.$$

Proof Let $X_{1},X_{2},\ldots ,X_{m}$ be a random sample from the standard laplace distribution with pdf $f_{X}(x)=\frac{1}{2}e^{-|x|},x\in R$ and characteristic function $\psi_{X}(t)=\frac{1}{1+t^{2}},t\in R$. Therefore, $\psi_{\sum_{i=1}^{m}X_{i}}(t)=\frac{1}{{\left(1+t^{2}\right)}^{m}}$. Now, using Theorem 1 and the fact that [15, Chapter 24]

$$f_{\sum_{i=1}^{m}X_{i}}(x)=\frac{e^{-|x|}}{2^{2m-1}}\sum_{j=0}^{m-1}\left(\begin{array}{c} 2m-j-2\\ m-1 \end{array}\right)\frac{{\left(2x\right)}^{j}}{j!},$$

we get

$$\int_{-\infty }^{\infty }\frac{e^{ity}}{{\left(1+t^{2}\right)}^{m}}dt=\int_{-\infty }^{\infty }e^{ity}\psi_{\sum_{i=1}^{m}X_{i}}(t)dt=2\pi f_{\sum_{i=1}^{m}X_{i}}(y)=2\pi \frac{e^{-|y|}}{2^{2m-1}}\sum_{j=0}^{m-1}\left(\begin{array}{c} 2m-j-2\\ m-1 \end{array}\right)\frac{{\left(2y\right)}^{j}}{j!}.$$

Hence, the characteristic function of the *t* distribution with 2m−1,m∈N degree of freedom is given by

(19) $$\psi_{T_{2m-1}}(t)=\int_{R}e^{itx}\frac{\Gamma (m)}{\sqrt{(2m-1)\pi }\Gamma (\frac{2m-1}{2})}{\left(1+\frac{x^{2}}{2m-1}\right)}^{-m}dx=\frac{\Gamma (m)}{\sqrt{\pi }\Gamma (\frac{2m-1}{2})}\int_{R}\frac{e^{i\sqrt{2m-1}tx}}{{\left(1+x^{2}\right)}^{m}}dx=\frac{2^{2m-2}{\left[(m-1)!\right]}^{2}}{\pi (2m-2)!}\times 2\pi \frac{e^{-|\sqrt{2m-1}t|}}{2^{2m-1}}\sum_{j=0}^{m-1}\left(\begin{array}{c} 2m-j-2\\ m-1 \end{array}\right)\frac{{\left(2\sqrt{2m-1}t\right)}^{j}}{j!}=e^{-|\sqrt{2m-1}t|}\sum_{j=0}^{m-1}\frac{\left(\begin{array}{c} 2m-j-2\\ m-1 \end{array}\right)}{\left(\begin{array}{c} 2m-2\\ m-1 \end{array}\right)}\frac{{\left(2\sqrt{2m-1}t\right)}^{j}}{j!}.$$

 □

Corollary 4 *From* Theorem 2*, the coefficient of the recurrence formula in* (17) *from Mitra* *[14]* *can be explicitly written as*

$$c_{j,m-1}=\frac{\left(\begin{array}{c} 2m-j-2\\ m-1 \end{array}\right)2^{j}}{\left(\begin{array}{c} 2m-2\\ m-1 \end{array}\right)j!}.$$

Next, we derive an explicit form for the pdf of linear combination of independent *t* random variables with odd degrees of freedom.

Theorem 3 *Let* $X_{1},X_{2},\ldots ,X_{n}$ *be independent t distributed random variables with* $2m_{i}-1,i=1,2,\ldots ,n,m_{i}\in N$ *degrees of freedom. Then the pdf of* $Y=\sum_{i=1}^{n}\alpha_{i}X_{i},\alpha_{i}\in R$ *is given by*

$$f_{Y}(y)=\frac{\sum_{j_{1}=0}^{m_{1}-1}\ldots \sum_{j_{n}=0}^{m_{n}-1}c_{j_{1},m_{1}-1}\ldots c_{j_{n},m_{n}-1}h(\alpha_{1}\sqrt{2m_{1}-1},j_{1},y)\ldots h(\alpha_{n}\sqrt{2m_{n}-1},j_{n},y)}{\sum_{j_{1}=0}^{m_{1}-1}\ldots \sum_{j_{n}=0}^{m_{n}-1}c_{j_{1},m_{1}-1}\ldots c_{j_{n},m_{n}-1}\frac{\Gamma (j_{1}+1)}{\left|\alpha_{1}\sqrt{2m_{1}-1}\right|}\ldots \frac{\Gamma (j_{n}+1)}{\left|\alpha_{n}\sqrt{2m_{n}-1}\right|}},$$

*where* h(γ,k,y) *is given in equation* (20)*.*

Proof From Theorem 2, we have

$$\psi_{Y}(t)=\prod_{i=1}^{n}\psi_{X_{i}}(t\alpha_{i})=\prod_{i=1}^{n}e^{-|\sqrt{2m_{i}-1}t\alpha_{i}|}\sum_{j_{i}=0}^{m_{i}-1}\frac{\left(\begin{array}{c} 2m_{i}-j_{i}-2\\ m_{i}-1 \end{array}\right)}{\left(\begin{array}{c} 2m_{i}-2\\ m_{i}-1 \end{array}\right)}\frac{{\left(2\sqrt{2m_{i}-1}t\alpha_{i}\right)}^{j_{i}}}{j_{i}!}=e^{-|t|\sum_{i=1}^{n}|\alpha_{i}\sqrt{2m_{i}-1}|}\sum_{j_{1}=0}^{m_{1}-1}\ldots \sum_{j_{n}=0}^{m_{n}-1}\frac{\left(\begin{array}{c} 2m_{1}-j_{1}-2\\ m_{1}-1 \end{array}\right)}{\left(\begin{array}{c} 2m_{1}-2\\ m_{1}-1 \end{array}\right)}\frac{{\left(2\sqrt{2m_{1}-1}t\alpha_{1}\right)}^{j_{1}}}{j_{1}!}\times \ldots \times \frac{\left(\begin{array}{c} 2m_{n}-j_{n}-2\\ m_{n}-1 \end{array}\right)}{\left(\begin{array}{c} 2m_{n}-2\\ m_{n}-1 \end{array}\right)}\frac{{\left(2\sqrt{2m_{n}-1}t\alpha_{n}\right)}^{j_{n}}}{j_{n}!}.$$

Now, from Theorem 1 we have $f_{Y}(y)=\frac{\int_{R}e^{ity}\psi_{Y}(t)dt}{\int_{R}\psi_{Y}(t)dt}$.

One can show that

$$\int_{R}e^{-|t\gamma |}|t\gamma {|}^{k}dt=\frac{2}{\left|\gamma \right|}\Gamma (k+1)$$

and

(20) $$\int_{R}e^{-|t\gamma |}|t\gamma {|}^{k}e^{ity}dt=2\frac{\Gamma (k+1)}{\left|\gamma \right|}\left[\frac{1+\sum_{w=k-1,k-3,\ldots ,(0\;\text{or}\;1)}\left(\begin{array}{c} k+1\\ w \end{array}\right){\left(\frac{iy}{\left|\gamma \right|}\right)}^{k+1-w}}{{\left(1+\frac{y^{2}}{\gamma^{2}}\right)}^{k+1}}\right]$$

The proof ends by setting $h(\gamma ,k,y)=\int_{R}e^{-|t\gamma |}|t\gamma {|}^{k}e^{ity}$, and noting that $c_{j,m-1}=\frac{\left(\begin{array}{c} 2m-j-2\\ m-1 \end{array}\right)2^{j}}{\left(\begin{array}{c} 2m-2\\ m-1 \end{array}\right)j!}$. □

## 4 Conclusion

Special case of weighted distributions with the corresponding characteristic functions have been proposed and discussed in this paper. The weighted distributions have the multimodal property with sine and cosine functions as their corresponding weight functions. Also, a method of calculating some interesting improper integrals is discussed in this paper including a closed form of $\int_{0}^{\infty }\frac{sin^{m}(x)}{x^{m}}dx$. Furthermore, closed form of the probability density function for linear combination of independent *t* distributed random variables with odd degrees of freedom is derived. The structural properties of the proposed multimodal weighted family of distributions can be studied in detail in future study.

## Declarations

### Author contribution statement

A. Alzaatreh, J. Kazempoor, A. Ahmadi Nadi: Conceived and designed the experiments; Performed the experiments; Analyzed and interpreted the data; Contributed reagents, materials, analysis tools or data; Wrote the paper.

### Funding statement

This work was supported by the 10.13039/501100002724American University of Sharjah Open Access Program.

### Competing interest statement

The authors declare no conflict of interest.

### Additional information

No additional information is available for this paper.
